# Complete Genome Sequence of Brucella abortus 68, Isolated from Aborted Fetal Sheep in Ukraine

**DOI:** 10.1128/MRA.01436-20

**Published:** 2021-03-11

**Authors:** Vitaliy Bolotin, Ganna Kovalenko, Nataliia Marchenko, Oleksii Solodiankin, Nataliia Rudova, Valentina Kutsenko, Eric Bortz, Anton Gerilovych, Devin M. Drown

**Affiliations:** a National Scientific Center Institute of Experimental and Clinical Veterinary Medicine (NSC IECVM), Kharkiv, Ukraine; b National Academy of Agrarian Sciences of Ukraine, Institute for Veterinary Medicine (IVM), Kyiv, Ukraine; c Department of Biological Sciences, University of Alaska Anchorage, Anchorage, Alaska, USA; d Department of Biology and Wildlife, University of Alaska Fairbanks, Fairbanks, Alaska, USA; e Institute of Arctic Biology, University of Alaska Fairbanks, Fairbanks, Alaska, USA; Loyola University Chicago

## Abstract

The complete genome sequence of Brucella abortus 68, isolated from an aborted sheep fetus in Luhansk, Ukraine, was assembled using Nanopore sequences. Two circular chromosomes totaling 3,281,317 bp (*N*_50_, 2,124,943 bp) comprised the complete genome sequence. The strain encodes the fosfomycin antibiotic resistance gene *fosX*, highlighting the risk of cross-species livestock and human infection.

## Abstract (precis)

The complete genome sequence of Brucella abortus 68, isolated from an aborted sheep fetus in Luhansk, Ukraine, was assembled using Nanopore sequences. Two circular chromosomes totaling 3,281,317 bp (*N*_50_, 2,124,943 bp) comprised the complete genome sequence. The strain encodes the fosfomycin antibiotic resistance gene *FosX*, highlighting the risk of cross-species livestock and human infection.

## ANNOUNCEMENT

As part of a scientific initiative to enhance veterinary diagnostic capacity in Ukraine, we sequenced the genome of a *Brucella* sp. strain isolated in 1974 from the Luhansk region in Ukraine. This isolate is part of a historical collection of bacterial livestock pathogens archived at the National Scientific Center Institute of Experimental and Clinical Veterinary Medicine in Ukraine. The sequencing of this strain using the Oxford Nanopore Technologies (ONT) MinION platform in veterinary laboratories in Ukraine represents a genomic exploration of this collection and will provide insight into circulating livestock pathogens.

The abomasum content of an aborted sheep’s fetus was added to meat-peptone-liver-glucose-glycerol (MPLGG) broth at 37°C overnight and then plated onto MPLGG agar. All media contained 20% bovine serum. A single colony was taken for pure culture. The isolated strain was lyophilized and stored until revival for sequencing in 2019. To revive the lyophilized strain, we added MPLGG broth without serum and then plated it onto MPLGG agar. For DNA extraction, we isolated a single colony by loop and added it to lysing buffer as input for the DNeasy UltraClean microbial kit (Qiagen).

We used 1 μg of DNA as input for a rapid sequencing library (SQK-RAD004; ONT) and sequenced it on an R9.4.1 flow cell (FLO-MIN106; flow cell ID FAK90503) for 48 h using a MinION Mk1B device. We base called the raw data using Guppy v4.2.2 (ONT) using the high accuracy model (-c dna_r9.4.1_450bps_hac.cfg) and default parameters. This run generated 11,677,814,462 bp in 3,163,635 reads with an average read length of 3,691 bp. We used Filtlong v0.2.0 (https://github.com/rrwick/Filtlong) to filter reads ≤50 bp long (--min_length 50) and with a Q score of ≤10 (--min_mean_q 90). After filtering, we had 8,786,405,255 bp in 2,101,589 reads with a read length *N*_50_ of 7,747 bp and a median Q score of 13.0.

We *de novo* assembled the genome sequence using Flye v2.81 (1) using the 8.8-Gb quality-controlled data set (coverage, 2,745×), specifying Nanopore reads (-nanopore-raw). To decrease the assembly time, we subsampled for initial disjointing assembly (--asm-coverage 100), which requires specifying an estimated genome size (--genomesize = 3.2m). Our draft assembly contained two contigs identified as circular by Flye. We corrected the assembly using 2 rounds of Racon v1.4.19 (2) polishing with the following parameters: score for matching bases (--match 8), score for mismatching bases (--mismatch -6), threshold for average base quality of windows (--quality-threshold -1), default gap penalty (--gap -8), and default window (--window-length 500). We ran a final polish with Medaka v1.1.3 (https://github.com/nanoporetech/medaka), specifying the base-caller model (-m r941_min_high_g360) and using default parameters. Our 3,281,317-bp (*N*_50_, 2,124,943 bp) polished assembly consists of two circular contigs (GC content, 57.21%). We used Circlator v1.5.5 (3) to rotate the polished assembly and fix the start positions.

During the data submission pipeline, the two chromosomes of the genome deposited in GenBank were annotated with PGAP v5.0 (4) and contain 55 tRNAs, 9 rRNAs, and 3,329 coding DNA sequences (CDS). PATRIC v3.6.7 (5, 6) further identified a total of 34 antibiotic resistance gene copies (Table 1) and 228 virulence factors. PATRIC also reported a 100% completeness score with no contamination (0%).

**TABLE 1:** Antimicrobial resistance genes

Antimicrobial resistance mechanism	Gene(s)
Antibiotic inactivation enzyme	*fosX*
Antibiotic target in susceptible species	*alr*, *ddl*, *EF-G*, *EF-Tu*, *folA*, *dfr*, *folP*, *gyrA*, *gyrB*, *inhA*, *fabI*, *Iso-tRNA*, *kasA*, *murA*, *rho*, *rpoB*, *rpoC*, *S10p*, *S12p*
Efflux pump conferring antibiotic resistance	*macA*, *macB*, *triABC-opmH*
Gene conferring resistance via absence	*gidB*
Protein altering cell wall charge conferring antibiotic resistance	*gdpD*, *pgsA*
Regulator modulating expression of antibiotic resistance genes	*oxyR*

We used the mlst command line tool (https://github.com/tseemann/mlst) to compare our genome sequence with public multilocus sequence typing schemes on PubMLST (7; https://pubmlst.org/organisms/brucella-spp). We found the isolate to have sequence type 2 (*gap-2*, *aroA-1*, *glk-2*, *dnaK-2*, *gyrB-1-23*, *trpE-3*, *cobQ-1*, *int_hyp-1*, *omp25-1*). By complete genome distance with a comparison of reference and representative genome sequences on PATRIC using Mash (8), this genome sequence is most similar to that of B. abortus 9-941 (GenBank accession no. GCF_000008145.1), isolated from cattle (9). Using *in silico* multiple-locus variable-number tandem-repeat analysis (MLVA), we manually searched the position of the MLVA-16 panel primer pairs (10, 11) in the full-genome sequence. We compared the length of each tandem-repeat locus with data from the *Brucella* table for allele assignment in MLVAbank (http://mlva.i2bc.paris-saclay.fr/mlvav4/genotyping/). Using these MLVA-16 data (2-5-3-12-2-2-3-1-6-41-8-4-9-4-3-3), Brucella abortus 68 is most similar to B. abortus BfR 124 bv. 6, isolated from cattle in Syria in 1993.

### Data availability.

This whole-genome project has been deposited in GenBank under accession no. CP066175 and CP066176. The versions described in this paper are the first versions, CP066175.1 and CP066176.1. The raw data for this project can be found in the SRA under accession no. PRJNA685163.

## References

[1] Kolmogorov M, Yuan J, Lin Y, Pevzner PA. 2019. Assembly of long, error-prone reads using repeat graphs. Nat Biotechnol 37:540–546. doi:10.1038/s41587-019-0072-8. 30936562

[2] Vaser R, Sovic I, Nagarajan N, Sikic M. 2017. Fast and accurate de novo genome assembly from long uncorrected reads. Genome Res 27:737–746. doi:10.1101/gr.214270.116. 28100585 PMC5411768

[3] Hunt M, De Silva N, Otto TD, Parkhill J, Keane JA, Harris SR. 2015. Circlator: automated circularization of genome assemblies using long sequencing reads. Genome Biol 16:294. doi:10.1186/s13059-015-0849-0. 26714481 PMC4699355

[4] Tatusova T, DiCuccio M, Badretdin A, Chetvernin V, Nawrocki EP, Zaslavsky L, Lomsadze A, Pruitt KD, Borodovsky M, Ostell J. 2016. NCBI Prokaryotic Genome Annotation Pipeline. Nucleic Acids Res 44:6614–6624. doi:10.1093/nar/gkw569. 27342282 PMC5001611

[5] Wattam AR, Davis JJ, Assaf R, Boisvert S, Brettin T, Bun C, Conrad N, Dietrich EM, Disz T, Gabbard JL, Gerdes S, Henry CS, Kenyon RW, Machi D, Mao C, Nordberg EK, Olsen GJ, Murphy-Olson DE, Olson R, Overbeek R, Parrello B, Pusch GD, Shukla M, Vonstein V, Warren A, Xia F, Yoo H, Stevens RL. 2017. Improvements to PATRIC, the all-bacterial Bioinformatics Database and Analysis Resource Center. Nucleic Acids Res 45:D535–D542. doi:10.1093/nar/gkw1017. 27899627 PMC5210524

[6] Brettin T, Davis JJ, Disz T, Edwards RA, Gerdes S, Olsen GJ, Olson R, Overbeek R, Parrello B, Pusch GD, Shukla M, Thomason JA, III, Stevens R, Vonstein V, Wattam AR, Xia F. 2015. RASTtk: a modular and extensible implementation of the RAST algorithm for building custom annotation pipelines and annotating batches of genomes. Sci Rep 5:8365. doi:10.1038/srep08365. 25666585 PMC4322359

[7] Jolley KA, Bray JE, Maiden MCJ. 2018. Open-access bacterial population genomics: BIGSdb software, the PubMLST.org website and their applications. Wellcome Open Res 3:124. doi:10.12688/wellcomeopenres.14826.1. 30345391 PMC6192448

[8] Ondov BD, Treangen TJ, Melsted P, Mallonee AB, Bergman NH, Koren S, Phillippy AM. 2016. Mash: fast genome and metagenome distance estimation using MinHash. Genome Biol 17:132. doi:10.1186/s13059-016-0997-x. 27323842 PMC4915045

[9] Halling SM, Peterson-Burch BD, Bricker BJ, Zuerner RL, Qing Z, Li LL, Kapur V, Alt DP, Olsen SC. 2005. Completion of the genome sequence of Brucella abortus and comparison to the highly similar genomes of Brucella melitensis and Brucella suis. J Bacteriol 187:2715–2726. doi:10.1128/JB.187.8.2715-2726.2005. 15805518 PMC1070361

[10] Le Flèche P, Jacques I, Grayon M, Al Dahouk S, Bouchon P, Denoeud F, Nöckler K, Neubauer H, Guilloteau LA, Vergnaud G. 2006. Evaluation and selection of tandem repeat loci for a Brucella MLVA typing assay. BMC Microbiol 6:9. doi:10.1186/1471-2180-6-9. 16469109 PMC1513380

[11] Al Dahouk S, Le Flèche P, Nöckler K, Jacques I, Grayon M, Scholz HC, Tomaso H, Vergnaud G, Neubauer H. 2007. Evaluation of Brucella MLVA typing for human brucellosis. J Microbiol Methods 69:137–145. doi:10.1016/j.mimet.2006.12.015. 17261338

